# Zeit-bezogene Perspektiven auf Jugend und Erfahrungen von Jugendlichen in der Corona-Pandemie

**DOI:** 10.1007/s11612-022-00623-y

**Published:** 2022-02-22

**Authors:** Lea Heyer

**Affiliations:** Institut für Sozial- und Organisationspädagogik, Stiftung Universität Hildesheim, Universitätsplatz 1, 31141 Hildesheim, Deutschland

**Keywords:** Jugend, Corona, Zeit, Biografie, Optimierung, Peer-Beziehungen, Youth, COVID-19, Time, Biography, Optimization, Peer relations

## Abstract

Dieser Beitrag der Zeitschrift Gruppe. Interaktion. Organisation. (GIO) thematisiert die Erfahrungen, die Jugendliche in Deutschland während der Corona-Pandemie gemacht haben, aus einer Zeit-bezogenen Perspektive. Dazu wird ein Verständnis von Zeit angelegt, mit welchem Biografien als gesellschaftlich geordnete Strukturmuster verstanden werden. Im ersten Teil werden jugendtheoretische Überlegungen aus der Entwicklungspsychologie, Erziehungswissenschaft und Soziologie mit Blick auf Charakteristika präsentiert, welche Jugend als Zeit besonderer Veränderungen und Entwicklungsaufgaben kennzeichnen. Dabei stehen somatische Veränderungen, der Bedeutungszuwachs von Peer-Beziehungen und Gruppenerfahrungen sowie die Konzeptionalisierung von Jugend als gesellschaftlich vorstrukturierte ‚Bildungszeit‘ im Fokus. Diese Ausführungen stellen den Rahmen für den zweiten Teil des Artikels dar. In diesem werden Ergebnisse aus den „JuCo“-Studien I und II, die vom Forschungsverbund „Kindheit – Jugend – Familie in der Corona-Zeit“ der Universitäten Hildesheim und Frankfurt a. M. durchgeführt wurden, präsentiert. Es wird mit Kommentaren gearbeitet, welche Befragte am Ende eines Online-Fragebogens freiwillig hinterlassen haben. Die Berichte der Jugendlichen weisen auf Erfahrungen von Frustration über verpasste Chancen, Leistungsdruck und Zukunftsängste unter den jungen Menschen hin. Die Implikationen dieser Befunde werden abschließend mit Blick auf Bedarfe für die Gestaltung der Zeit nach der Pandemie diskutiert. Der Beitrag schließt mit einer Forderung nach offenen sozialen Räumen, in denen Jugendliche die Möglichkeit zu intergenerationalen und peer-bezogenen Begegnungen haben.

## Abstract (en)

This paper in the journal Gruppe. Interaktion. Organisation. (GIO) addresses the experiences of young people in Germany during the corona pandemic from a time-related perspective. For this, it draws on conceptualisations of time through which biographies can be understood as socially ordered structural patterns. First, theoretical reflections on youth are presented, focusing on psychological, educational and sociological perspectives with regards to characteristics that mark youth as a time of specific biographical changes. This is centred on somatic changes, the growing importance of peer relations and group experiences, as well as the implications of youth as a socially pre-structured time for education. These considerations provide the framework for the second part of the article. Results from the “JuCo” studies, undertaken by the research group “childhood—youth—family during times of COVID-19” at the Universities of Hildesheim and Frankfurt a. M., are presented. The data used are comments that respondents made at the end of an online questionnaire. The quotations indicate experiences of frustration about missed opportunities, increased performance pressure, and fears about the future among young people. Implications of these observations are discussed against the backdrop of needs and requirements for the time after the pandemic. The article closes with a summary, outlining the demand for creating open social spaces that foster opportunities for intergenerational and peer-related encounters among young people.

## Einleitung

Die Corona-Pandemie bedeutet für sehr viele Menschen inzwischen seit etwa zwei Jahren eine Ausnahmesituation. Das Leben von Jugendlichen veränderte sich unter anderem durch die Umstellung auf Online-Unterricht in der Schule, Berufsschule oder Universität deutlich. Aber auch die Schließung von Freizeitangeboten sowie die unterschiedlichen Kontaktbeschränkungen bedeuteten für viele junge Menschen^1^ grundlegende Veränderungen. So machten viele während der Pandemie Erfahrungen von Frustration und Einsamkeit (Andresen et al. 2021). Immer wieder wird in Diskussionen über die Auswirkungen der Pandemie die Position vertreten, dass die Maßnahmen zur Einschränkung des Infektionsgeschehens unterschiedliche gesellschaftliche Gruppen unterschiedlich betreffen. Dabei wird nicht nur mit Blick auf die Infektionsrisiken der „über 60-Jährigen“ (RKI 2020), sondern auch in anderen Zusammenhängen u. a. auf unterschiedliche Altersgruppen Bezug genommen. So wird beispielsweise oft gesagt, dass ‚ein Jahr Pandemie‘ im Lebens einer 16-Jährigen nicht das gleiche sei wie ein Jahr im Leben eines bspw. 40-Jährigen. Doch auf welche Annahmen bezieht sich diese Aussage eigentlich genauer?

Von dieser Frage ausgehend möchte ich einige Gedanken zur Diskussion stellen. Ich bringe dazu empirische Beobachtungen aus den „Jugend und Corona“ (JuCo)-Studien (Andresen et al. 2020a, b) zum Zeiterleben Jugendlicher in der Corona-Pandemie mit Beobachtungen zur Bedeutung von „Zeit“ für verschiedene Perspektiven auf „Jugend“ zusammen. In einem Ausblick stelle ich weiterführende Überlegungen zu möglichen Implikationen u. a. für die Organisation von generationenübergreifenden Bildungs- und Austauschgelegenheiten in post-pandemischen Zeiten an.

## Zum Zeitbegriff

Die Frage, was „Zeit“ ist, hat große Denker:innen schon mindestens seit der Antike beschäftigt und stellt einen wichtigen Gegenstand in fast allen seither entstandenen Wissenschaften dar: „Aus naturwissenschaftlicher Perspektive etwa ist Zeit eine physikalische, objektive und selbstverständlich berechenbare Größe. Aus erziehungs- und bildungswissenschaftlicher Perspektive zeigt sich das Phänomen Zeit als eine inhärente Ordnungskategorie von Lern- und Bildungsprozessen; betrachten wir das Individuum, dann ist Zeit schließlich eine sehr flexible und dehnbare Größe und unterliegt individuellen Wahrnehmungen, lebensgeschichtlichen Entwicklungsprozessen, bedeutsamen Erfahrungen und eigenen Vorlieben.“ (vgl. Mikula 2020, S. 3).

*Eine* Perspektive auf Zeit kann somit die Betrachtung des „biografisch-lebensgeschichten Weg[s] eines Subjekts [sein], der beeinflusst ist von natürlichen, kulturellen und gesellschaftlichen Zeitkonzepten und -zyklen“ (Mikula 2020). Mit Mikula (2020, S. 2) ist davon auszugehen, dass das Zeitbewusstsein von Individuen eingebettet ist in „individuelle, gesellschaftliche, kulturelle und zeitliche Lebenslaufstrukturen“ und dass „Lebenspraktiken in der Zeit […] je nach Lebensphase, in der sich Menschen befinden, einzigartige Zeitzüge und Zeitbezüge [enthalten].“ (Mikula 2020, S. 6).

Aus einer solchermaßen soziologisch inspirierten Sicht auf Zeit fokussiert der vorliegende Artikel auf Besonderheiten der Bedeutung von Zeit für das Verstehen von „Jugend“ als gesellschaftliche Bezugsgröße, insbesondere während der Corona-Pandemie. Zunächst sind jedoch ergänzend zum Begriff der Zeit einige Worte zum Jugendbegriff zu verlieren.

## Zum Jugendbegriff

Mit dem Begriff der „Jugend“ wird je nach disziplinärer Sichtweise Unterschiedliches beschrieben: „eine biologische Reifungsphase, ein Möglichkeitsraum der Entwicklung, eine Erziehungsaufgabe, ein juristischer Terminus und vieles mehr.“ (Harring et al. 2015, S. 12) Die jeweils vorgenommene Definition von Jugend bestimmt natürlich auch, mit Blick auf welche Aspekte für die Bestimmung dieser Lebens- und Entwicklungsphase Zeit eine Rolle spielt.

Über die Tatsache einer Fülle an unterschiedlichen Definitionen von Jugend hinaus ist festzuhalten, dass Jugend „sich auch höchst unterschiedlich aus[prägt]. Ein generalisierendes und einheitliches Bild lässt sich längst nicht mehr zeichnen.“ (Harring et al. 2015, S. 12) Bereits seit den 1990er-Jahren wird festgestellt, dass „die einheitliche kollektive Statuspassage Jugend in plurale Verlaufsformen und Zeitstrukturen […] [zerfällt]; es entwickeln sich gleichsam mehrere ‚Jugenden*‘*, die sich voneinander so stark unterscheiden, daß sie nicht mehr in einem Modell zusammengefaßt werden können“ (vgl. Harring et al. 2015, S. 12). Diese Ausdifferenzierung von Jugend „führt dazu, dass inzwischen kein einheitlicher Abschluss dieser Phase in Form einer Altersbegrenzung möglich ist“ (vgl. Harring et al. 2015, S. 13). Die Jugendphase „zeichnet sich durch viele Ungleichzeitigkeiten und asynchrone Entwicklungen aus […].“ (ebd.) Dies geht u. a. mit einer starken Pluralisierung von Lebensverläufen einher.

Man kann Jugend insofern nicht pauschal betrachten. Diese Perspektive soll bei der Einordnung der folgenden Ausführungen mitlaufen – denn auch wenn mit Mikula (2020, S. 3) davon ausgegangenen werden kann, dass „jedes Lebensalter […] sein subjektives Zeitempfinden“ hat, bleibt es aus jugendtheoretischer Sicht doch so, dass es eine bestimmte Qualität bspw. von Zeiterleben, die gewissermaßen ‚alle Jugendlichen‘ teilen, nicht geben kann. Gleichzeitig sind trotz dieser Heterogenität einige querliegende Aspekte zu beobachten, die ich im Folgenden aus verschiedenen disziplinären Perspektiven skizzieren möchte.

## Zeit-bezogene Perspektiven auf Jugend aus verschiedenen Disziplinen

Im Versuch, das Wesen und die Eigenarten von Jugend verstehen, werden häufig psychologische, erziehungswissenschaftliche und sozialpädagogische sowie soziologische Perspektiven unterschieden. So kann Jugend beispielsweise als biologisches Lebensalter, biografische Lebensphase oder als gesellschaftliche Institution in den Blick genommen werden. Im Folgenden orientiere ich mich an dieser disziplinären Strukturierung und möchte Überlegungen zur Bedeutung von Zeit für das jeweilige Verständnis von Jugend anstellen.

### Jugend als Zeit somatischer Veränderungen

Aus einer entwicklungspsychologischen Perspektive auf Jugend stellt sich zunächst die Frage, wo bzw. wann Jugend beginnt und endet. Dies wird in der Entwicklungspsychologie vorrangig mit Blick auf physische und psychische Entwicklungsprozesse beschrieben. So führen bspw. Weichold und Silbereisen (2017, S. 240) aus: „Als Jugend im Sinne der Entwicklungspsychologie bezeichnet man die Zeit zwischen der Pubertät und dem Ende des zweiten Lebensjahrzehnts (ca. 10.–20. Lebensjahr).“ Jugend ist hier somit vor allem als Jugend*alter* und hierbei als Zeit der Pubertät zu verstehen. Diese ist gekennzeichnet durch grundlegende körperliche Veränderungen der Heranwachsenden (vgl. Weichold und Silbereisen 2017, S. 242). Die Dichte an Lern- und Entwicklungsschritten ist in der Jugendphase im Gegensatz zum Erwachsenenalter besonders hoch, d. h. es vollziehen sich deutlich mehr Entwicklungen und dies in kürzerer Zeit (Freund und Nikitin 2017, S. 266).

Der Faktor Zeit ist aus entwicklungspsychologischer Perspektive auf Jugend insbesondere dort interessant, wo bspw. somatische Veränderungen mit sozialen Entwicklungen in Verbindung gebracht werden. So referieren Weichold und Silbereisen (2017, S. 244) etwa die Beobachtung, dass sich die Pubertät in westlichen Ländern „aufgrund besserer Ernährung und Hygiene nach vorn verschoben [hat]“ und bringen diese ins Verhältnis zu der Tatsache, dass gleichzeitig jugendtypische „Entwicklungsaufgaben […] mehr Zeit [beanspruchen], weil die Anforderungen komplexer und ihre Planbarkeit geringer“ geworden seien (Weichold und Silbereisen 2017, S. 250). Aus dieser Beobachtung ergibt sich eine „besondere Vulnerabilität“ von Jugendlichen bei der Entwicklung von Selbstregulationsfähigkeiten in Bezug auf das eigene Handeln (vgl. Weichold und Silbereisen 2017, S. 250). Das bedeutet, dass Jugendliche beim „Erkennen von Diskrepanzen zwischen Wunsch, Realität und Umsetzung“ (ebd.) ihrer Lebensaspirationen auf besondere Weise verletzlich sind. Vor diesem Hintergrund könnte sich eine besondere Vulnerabilität von Jugendlichen auch bzgl. der Einschnitte ergeben, die die Corona-Pandemie für ihr Leben mit sich bringt.

Auch mit Blick auf die benötigte Zeit für Lernerfolge im Kontext Schule sind die entwicklungspsychologischen Erkenntnisse folgenreich. „[D]as biologische Entwicklungstempo und der Stand der kognitiven Entwicklung“ etwa können sich „zwischen Jugendlichen des gleichen Jahrgangs erheblich unterscheiden“ (Weichold und Silbereisen 2017, S. 257). Hieraus ergibt sich, dass die Lernumgebung Jugendlicher auf diese Unterschiede äußerst flexibel eingehen muss bzw. müsste, insofern eine Passung erreicht werden soll (ebd.). In der Corona-Zeit ist jedoch wahrscheinlich, dass im Zuge bspw. von Homeschooling, Wechselunterricht und Online-Studium noch deutlich weniger auf die individuellen Lernvoraussetzungen und -tempi der Jugendlichen eingegangen werden konnte.

### Jugend als Zeit der Veränderung sozialer Kontakte

Mit Blick auf die psycho-soziale Dimension von Jugend sind aus einer Zeit-bezogenen Perspektive bspw. die Freizeitgestaltung und die Rolle von Peers interessant. In der Jugend nehmen Peer-Beziehungen, d. h. Kontakte unter Gleichaltrigen, zu und lösen damit häufig die Familie als bis dato primäres soziales Bezugssystem ab (vgl. Harring et al. 2015, S. 24f). In der Freizeit ebenso wie in der Schule entstehen damit neue Bildungs- und Sozialisationsräume, die „in besonderem Maße auf die (kulturelle) Lebensführung und soziale Orientierung von Jugendlichen“ wirken (ebd.).

Hier kann festgehalten werden, dass die Jugend eine Lebensphase ist, in welcher junge Menschen lernen und üben, autonomer über die Art zu entscheiden, wie sie ihre Zeit verbringen. So zeigt sich bei der Betrachtung alterstypischer Verläufe des Freizeitverhaltens Jugendlicher nach (vgl. Weichold und Silbereisen 2017, S. 256) das folgende Muster: Zunächst verbringen Jugendliche ihre Freizeit in einer für sie von Erwachsenen organisierten Form („organized“). Durch den Bedeutungsgewinn von informellen Beziehungen zu Peers werden die Aktivitäten zunehmend selbst- und weniger fremdgesteuert („casual“, „informal“). Mit wachsender Selbstständigkeit (auch finanzieller) werden schließlich vermehrt kommerziell gestaltete Freizeitangebote in Anspruch genommen („commercial“). Die Maßnahmen zur Eindämmung des Corona-Virus haben diese Entwicklung jedoch deutlich eingeschränkt. So dürften sich etwa die Möglichkeiten, Peer-Beziehungen als Lernräume zu nutzen, stark verringert haben.

Gleichzeitig ist anzumerken, dass die Gestaltungsspielräume von Jugendlichen bezogen auf ihre Freizeit sehr unterschiedlich sind (vgl. Harring et al. 2015, S. 25). Nicht alle jungen Menschen haben gleichermaßen die Chance, sich entsprechend ihren „jugendlichen Motiven, die sich zwischen Abenteuerlust, Unabhängigkeitsbestreben und Qualifizierungsabsichten bewegen“, in ihrer Freizeit sowie in ihrer Lebensplanung national und international mobil zu bewegen und neue Beziehungen einzugehen (vgl. Harring et al. 2015, S. 26). Hinweise auf eine Verstärkung von sozialen Ungleichheiten durch die Pandemie (BJK 2021) legen nahe, dass sich diese Unterschiede in den letzten zwei Jahren noch verstärkt haben dürften.

### Jugend als biografisch angelegte Bildungszeit

Durch die Aufteilung von Biografien in Lebensphasen stehen uns diese als „sozialweltliches Orientierungsmuster“ zur Verfügung, „das in modernen Gesellschaften zum Alltagswissen gehört und das wir als Ordnungsschema für unser Handeln und Denken nutzen.“ (Dausien 2020, S. 73) Mit jeder Lebensphase gehen „lebensalterbezogene Anforderungen“ einher, die „gleichermaßen institutionell wie individuell disponiert sind“ (Schierbaum und Bossek 2020, S. 191). Die gesellschaftlich vorstrukturierten, alterstypischen Erwartungen an Jugend sind aus soziologischer Sicht als historisch bedingt und wandelbar zu verstehen. Die Lebensphase Jugend wird als erst durch die „Institutionalisierung eines dreigeteilten modernen Lebenslaufes“, welcher „um Erwerbsarbeit strukturiert ist“ (vgl. Dahmen 2020, S. 174) entstanden begriffen. Zentral sind dabei Prozesse der Dekommodifizierung und Scholarisierung sowie der Institutionalisierung einer Altershierarchie (Dahmen 2020, S. 174).

Ein wichtiges Konzept ist hier jenes des „psychosozialen Moratoriums“. Jugend wird als institutionalisierter „Schonraum“ verstanden, in dem „Menschen zeitlich befristet von bestimmten gesellschaftlichen Verpflichtungen ent[bunden]“ werden (Harring et al. 2015, S. 15 mit Bezug auf Reinders 2006, S. 82). Das Konzept wurde mit Blick auf die in den 1970er-Jahren einsetzende Bildungsexpansion ausdifferenziert und bezeichnet heute zumeist den Prozess der „Etablierung eines sich über die gesamte Lebensphase Jugend erstreckenden Bildungsmoratoriums […] mitsamt dem daraus resultierenden Trend zu längeren Ausbildungszeiten und höheren schulischen Qualifikationen“ (Harring et al. 2015, S. 16). Damit einher geht die gesellschaftliche Erwartung an Jugendliche, diese „Freistellung auf Zeit“ zu nutzen, um „sich kulturelles Kapital allgemein und Bildungskapital in Form von Bildungstiteln im Besonderen anzueignen“ (vgl. Harring et al. 2015, S. 16). Der Übergang ins Erwachsenenalter soll durch Leistung vorbereitet, das Bildungsmoratorium ziel- und zweckgerichtet ausgestaltet werden (Harring et al. 2015, S. 16).

Eine Aktualisierung hat das Moratoriums-Konzept zuletzt im Lichte gesellschaftlicher Prozesse der Beschleunigung und Individualisierung erfahren, sodass inzwischen gar von Jugend als „Optimierungsmoratorium“ gesprochen wird (vgl. Reinders 2016). Hintergrund ist, dass sich „[u]nter den Bedingungen von Bildung und Qualifizierung […] im ökonomischen Wettbewerb und demografischen Wandel […] Leistungs- und Optimierungsanforderungen zunehmend in frühe Phasen des Lebens“ vorverlagern (Schierbaum und Bossek 2020, S. 192). Gleichzeitig hat „die den Individuen zugeschriebene Verantwortung für ihren Lebenslauf zugenommen, indem diese sich zugleich als ‚unternehmerisch‘ und ‚singulär‘ ausweisen müssen, um ihre Kompetenz der Lebensgestaltung zu zeigen“ (vgl. Sackmann 2020, S. 186). Es ist daher zu vermuten, dass Jugendliche eventuelle Corona-bedingte Einschnitte in ihre Zukunftsplanung sowie in ihre avisierten Bildungskarrieren als Gefahr für ihren in diesem Sinne ‚unternehmerischen‘, hoch-individualisierten Bildungserfolg wahrnehmen könnten.

## Zeiterleben von Jugendlichen in der Corona-Zeit

Wie im vorigen Abschnitt zusammengetragen, geraten mit einer Zeit-bezogenen Perspektive auf Jugend Aspekte in den Blick, die auch die Erfahrungen von Jugendlichen während der Pandemie in besonderer Weise prägen dürften. Ich möchte neben die soeben präsentierten Überlegungen daher nun einige Stimmen von Jugendlichen stellen. Dabei nehme ich Bezug auf Ergebnisse der „Jugend und Corona (JuCo)“ Studien I und II, die im Frühjahr und Herbst 2020 vom Forschungsverbund „Kindheit – Jugend – Familie in der Corona-Zeit“ durchgeführt wurden.

An den Studien beteiligten sich über 5520 (JuCo I) bzw. über 7000 (JuCo II) junge Menschen, um in einem Online-Fragebogen Auskunft über ihre Erfahrungen in der Pandemie zu geben (Andresen et al. 2021, 2020a, b). Die Befragten waren zwischen 15 und 30 Jahre alt, bewegen sich also aus einer Lebensphasen-bezogenen Perspektive zwischen Jugend und jungem Erwachsenenalter.

Während an anderen Stellen bereits vertieft auf die statistischen Ergebnisse der Studien eingegangen wurde (z. B. Lips et al. 2022 i.E., Heyer 2021), möchte ich mich hier auf die exemplarische Darstellung von Kommentaren konzentrieren, wie sie sehr viele Befragte am Ende des Fragebogens in einem offenen Kommentarfeld hinterließen. Ich möchte damit drei wesentliche Beobachtungen zum Zeiterleben von Jugendlichen in der Pandemie herausstellen. Dies sind: eine Frustration über verpasste Chancen, Berichte über Stress und Leistungsdruck, sowie die Schilderung von Zukunftsängsten.

### Frustration über „verpasste Chancen“

In vielen der Kommentare äußern Befragte der JuCo-Studien ihren Frust über Reisen, Unternehmungen, Treffen und besondere Erfahrungen, die sie aufgrund der Pandemie nicht antreten oder machen konnten. So etwa in den folgenden drei Schilderungen:^2^

Die Generation, die am Ende der Schule bis Ende des Studiums steht, leidet am meisten unter Corona. Das sind so kostbare Jahre. Man kann nicht einfach sagen „dieses Auslandspraktikum mache ich dann eben in einem Jahr“, weil eben nur jetzt dafür Zeit gewesen wäre im Studium (oder nach dem Abi).

Ich verbinde mit der Corona-Zeit Einsamkeit, Isolation und Stress. Ich bin im Abschlussjahrgang meiner Schule und viele Fahrten, auf die ich mich sehr gefreut habe, mussten ersatzlos gestrichen werden. Alles Schöne wurde uns genommen und kann auch nicht mehr nachgeholt werden, weil es ja unser letztes Jahr an der Schule ist.

Vor allem auf die neuen Bekanntschaften und das typische Studentenleben mit Partys, Vorlesungen auf dem Campus und die Mensa [hatte ich mich gefreut]. All das wurde mir durch Corona genommen. Jetzt sieht mein Alltag ziemlich monoton und langweilig aus: Aufstehen, Frühstück, vor den Laptop setzen, mir Vorlesungen anhören und mich in Tutorien möglichst beteiligen. Dabei sehe ich meistens nur den/die Professor:in oder den/die Dozent:in. Das nimmt den Spaß und das Persönliche aus dem Studium und ersetzt es mit reinem Stoff ohne das „Drum-und-Dran“.

Während die Erfahrung abgesagter Veranstaltungen etc. zunächst eine ist, die viele Mitglieder der Gesellschaft teilen, mischt sich in die Kommentare der Jugendlichen spürbar ein resignativer Tonfall. Hier wird von „kostbaren Jahren“ und unwiederbringlichen Zeitfenstern bspw. für ein Auslandspraktikum oder eine Abschlussfahrt mit dem Jahrgang gesprochen. Auch der Studienbeginn unter Corona-Bedingungen (hier offenbar im Modus der Online-Lehre) wird als herbe persönliche Enttäuschung erfahren, da neue Bekanntschaften deutlich schwieriger einzugehen sind und „das typische Studentenleben“ mit persönlichen Begegnungen z. B. auf dem Campus nicht erlebt werden kann.

### Leistungsdruck

Von sich aus nehmen zahlreiche Befragte der JuCo-Studien mit ihren Kommentaren Bezug auf ihre aktuelle schulische Situation bzw. ihre Erfahrungen in Ausbildung oder Universität. Dabei ist immer wieder die Rede von Überlastungen und Überforderung, insbesondere im Zusammenhang mit Online-Studium und Homeschooling. Einige Stimmen von Befragten der JuCo-Studien:

Durch Homeschooling lastet ein großer Druck auf mir, weil ich immer das Gefühl habe, nicht alles zu schaffen oder etwas zu verpassen, obwohl ich eine sehr gute Schülerin bin.

[Wir wurden] mit Aufgaben überladen. Ich saß teilweise bis zu 7 h an meinem Handy, weil das zu der Zeit mein einziges technisches Medium war, das ich im Homeoffice zur Verfügung hatte. Es war unglaublich viel, was ich für die Schule leisten musste. Viel mehr, als wenn man alles gemeinsam im Unterricht erarbeitet hätte.

Heute verbringe ich einen ganzen Tag im Online-Semester von 9:45 bis 21:30 und das laugt ziemlich aus. Auch die Gruppenarbeit, die nur noch online durchgeführt werden kann, ist anstrengend. Ich verbringe im Moment fast jeden Tag ausschließlich in Zoom Meetings, ob nun für eine Gruppenarbeit oder für die Vorlesung.

Ich verbinde mit der Corona-Zeit sehr viel Stress und Einsamkeit. Einsamkeit, weil ich meine Freunde nicht mehr wie gewohnt treffen kann und es auch das Leben in einer Fernbeziehung sehr erschwert […]. Gleichzeitig verlangt die Uni immer mehr. Sie verlangt wesentlich mehr als noch in den Semestern ohne Corona. Das bedeutet mehr Zuhause sitzen und lernen, aber weniger Abschalten und Ruhe.

Die Befragten schildern, dass die Aufgabe, ihr Lernen und ihren Alltag alleine und von zu Hause aus zu meistern, als schwierig empfunden wird. Es wird die Angst geäußert, mit dem Lernstoff nicht ‚hinterherzukommen‘. Außerdem ist von Druck, Stress und Erschöpfung die Rede, während Ruhe-Phasen sowie der Ausgleich durch Kontakte mit Peers selten geworden sind.

### Zukunftsängste

Nach ihrer Einstellung zur Zukunft wurden die Befragten explizit in einem Item des Fragebogens gefragt. Hierauf gaben im November 2020 über 45 % der Befragten an, voll bis eher zuzustimmen, Angst vor ihrer Zukunft zu haben (Andresen et al. 2020b, S. 7). Viele Kommentare illustrieren diesen Befund, wenn Jugendliche etwa schreiben:

Es ist super schwer mit Ausbildungs- oder Studienplätzen. […] Ich mache mir sehr starke Sorgen um meine Zukunft. Selbstverständlich geht es nicht nur mir so, aber wo soll das alles hinführen … Ich […] bin am Bewerbungen schreiben […] und jetzt durch Corona kamen mehr Absagen, oder auch Rückmeldungen wie: Wir können erst wieder ab 2022 Plätze anbieten. Es ist einfach sehr deprimierend und wie gesagt, man macht sich große Sorgen.

Mir selber geht es um die Ungewissheit und den Frust darüber, dass ja keiner etwas an der derzeitigen Situation ändern kann. Man muss einfach hinnehmen was vorgeschrieben wird. Aber ich mache mir schon Gedanken, inwiefern meine Berufswahl nach dem Abitur eingeschränkt sein wird.

Meinem Empfinden nach spaltet sich die Gesellschaft zurzeit in zwei Lager und davor habe ich sehr Angst, viel mehr als vor den wirklichen Maßnahmen und meinen persönlichen Möglichkeiten in der Zukunft, die sich durch Corona natürlich auch verändern können.

Ich glaube, viele junge Menschen können diese Phase durchaus überstehen, wenn sie auch als zeitlich begrenzte Phase dargestellt wird. Wirklich große Sorgen mache ich mir darum, ob ich es nun noch schaffe, ein Auslandssemester zu machen oder ob mir diese Möglichkeit für immer genommen wird. Mir fehlen Langzeitstrategien seitens der Politik.

Die jungen Menschen berichten über die Sorge, keinen passenden Ausbildungs- oder Studienplatz zu finden oder sich aufgrund eingeschränkter Möglichkeiten neu entscheiden zu müssen, wie die Berufswahl aussehen soll. Auch ist von der Angst zu lesen, dass die Pandemie noch deutlich länger anhalten und bspw. die Mobilität der jungen Menschen weiter einschränken könnte. Darüber hinaus äußern Befragte Sorgen bezogen auf gesellschaftliche Entwicklungen, wie etwa eine drohende ‚Spaltung der Gesellschaft‘. Deutlich wird insbesondere im letzten Zitat, dass der Eindruck besteht, dass diese Sorgen in der Politik nicht aufgegriffen werden. Dies korrespondiert mit dem Befund, dass im November 2020 fast 65 % der Befragten gar nicht bis eher nicht zustimmen, dass ihre Sorgen von der Politik gehört werden (Andresen et al. 2020b, S. 10).

## Diskussion

Ich möchte diese Stimmen junger Menschen nun mit den zuvor präsentierten Gedanken zur Bedeutung von Zeit für und in der Jugend zusammenbringen. Das Argument ist dabei, dass die Corona-Zeit für Jugendliche deshalb u. a. von Frustration über verpasste Chancen, Leistungsdruck und Zukunftsängste geprägt zu sein scheint, da ihr „subjektives Zeitempfinden“ (Mikula 2020, S. 3) in psychologischer und sozialer Hinsicht ein besonderes ist.

Wie beschrieben, ergibt sich aus den entwicklungspsychologischen und soziologischen Überlegungen die Annahme, dass Mitglieder einer Gesellschaft „Vorstellungen darüber teilen, welche Aufgaben […] zu einem bestimmten Alter verfolgt und erreicht werden sollten“ (Freund und Nikitin 2017, S. 269). Diese sozialen Erwartungen können in Anlehnung an das „social clock“-Modell von Neugarten (1972, zit. nach Freund und Nikitin 2017) als normativer Zeitplan des Lebens verstanden werden. Jugendliche wissen, dass eine Verletzung dieses Zeitplans zu negativen Sanktionen führt und richten sich deshalb in ihrer Lebensplanung und ihrer persönlichen Zielsetzung danach aus (ebd.). Können Ziele nicht wie gewünscht umgesetzt werden, kommt es normaler Weise zu einer Zielanpassung (Weichold und Silbereisen 2017, S. 250). Ähnlich wie z. B. in Phasen hoher Jugendarbeitslosigkeit ist dies in der Corona-Pandemie jedoch nicht ohne weiteres möglich. Dies könnte ein Aspekt sein, der auf Zukunftsunsicherheiten und Ängste verstärkend wirkt.

Hinzu kommt, dass Jugendliche heute höhere Bildungsaspirationen nicht nur erreichen *können*, sondern es geradezu *müssen*, „um eine günstige Ausgangsposition für ihren beruflichen Platzierungsprozess zu erhalten.“ (Harring et al. 2015, S. 18) „Eingebettet in Vergleichs- und Konkurrenzszenarien werden bestmögliche Leistungen in immer früheren Lebensphasen gefordert, während sich gleichzeitig Lerninhalte verdichten und in weniger Zeit bewältigt werden müssen.“ (Schreiber 2020, S. 234) Das ursprünglich als „Schonraum“ gedachte ‚Bildungsmoratorium Jugend‘ ist heute zunehmend „von Leistungsdruck und Entscheidungsnotwendigkeiten geprägt“ (ebd.). Dies kann insbesondere vor dem Hintergrund von Bildungsungleichheiten belastend sein. Die Erfahrung, im „positionalen Wettbewerb um Bildungszertifikate“ (Brown 2004) bspw. mit dem Pandemie-bedingten Homeschooling oder Online-Studium nicht ‚klarzukommen‘, kann den Stress und Leistungsdruck junger Menschen zusätzlich erhöhen.

Während etwa in der Shell Jugendstudie 2019 (zit. nach Köhler 2020, S. 367) hervorgehoben wird, dass Jugendliche ihre gesellschaftliche und persönliche Zukunft positiv einschätzen, weisen die Erfahrungen Jugendlicher in der Corona-Pandemie eine andere Färbung auf. In der ungewissen Situation der Pandemie ergeben sich neue Anforderungen an die Synchronisation von Zeitanforderungen mit deren Umsetzungsmöglichkeiten. Es ist nachvollziehbar, dass Jugendliche dies auch mit Blick auf die von ihnen vorgefundenen Angebote in Bildungsorganisationen und der Politik verlangen (vgl. Köhler 2020, S. 366). Sie reagieren mit Sorge und Unsicherheit angesichts der weitgehenden Abwesenheit von, wie es ein:e Befragte:r ausdrückt, „politischen Langzeitstrategien“ (s. oben) und Gelegenheiten, die eigene Meinung hörbar einzubringen.

## Fazit und Ausblick

Angesichts der angestellten Überlegungen sollen nun abschließend und weiterführend drei Gedanken dazu formuliert werden, welche Bedarfe sich aus den Beobachtungen ergeben könnten. Für die Gestaltung von Bildungsgelegenheiten in post-pandemischen Zeiten sind insbesondere Bildungsinstitutionen wie Schulen und Universtäten, aber gleichermaßen auch Arbeitgeber und Fachkräfte der (außerschulischen) Jugendarbeit ebenso wie unsereins im ganz alltäglichen Miteinander gefragt.

*Erstens* braucht es Gelegenheiten, um den der Pandemie innewohnenden Generationenkonflikt zwischen Jungen und Alten *miteinander* auszutragen. Der Austausch muss dabei über ein bloßes ‚Rücksichtnehmen‘ auf die „Alten“ und deren ‚Bemitleiden‘ der „Jungen“ hinausgehen. Jugendliche, Erwachsene und ältere Menschen müssen miteinander darüber reden, was die Pandemie für sie bedeutet (hat) und wie deren Folgen langfristig insbesondere für diejenigen, die jetzt jugendlich sind, aufgefangen und gesteuert werden können. Dafür müssen Aushandlungsräume geschaffen werden.

Hieraus ergibt sich direkt *zweitens*: Jugendliche müssen beteiligt werden. Sie müssen auch selbst diese Beteiligungsrechte einfordern und bestehende Mitbestimmungsmöglichkeiten nutzen. Wichtig ist aber auch, dass Politik und ältere Mitglieder der Gesellschaft bspw. in Familie und Schule die Sorgen und Bedarfe von Jugendlichen hören und einbeziehen. Dies darf nicht nur punktuell bei Fragen der Gestaltung von Maßnahmen zur Einschränkung der Pandemie erfolgen, sondern muss auch im Prozess der „Aufarbeitung“ und der Diskussion langfristiger Konsequenzen strukturell verlässlich organisiert werden.

*Drittens* braucht es dem vorgelagert für die Umsetzung dieser Forderungen mehr Räume für den Peer-Austausch unter Jugendlichen. Es müssen (wieder) Gelegenheiten geschaffen und gefördert werden, durch die sich Jugendliche über ihre Erfahrungen in der Pandemie austauschen können und gemeinsam Schlüsse ziehen können. Dies ist insbesondere wichtig, um sich von den durch die Pandemie noch verstärkten Individualisierungstendenzen, Leistungsdruck und Zukunftsängsten zu emanzipieren und sich ggf. überhaupt als Generation begreifen zu können.

Eine Orientierung für die Gestaltung solcher wenig strukturierter und gleichzeitig dichter Lern-Räume können gruppendynamische Lernformate und -methoden bieten. Diese könnten bspw. in der Zeit „nach der Online-Lehre“ in der Hochschule Erfahrungen ermöglichen, die einen Gegensatz zu Individualisierung und Optimierung bilden. So berichtet bspw. Lackner (2018, S. 364), dass insbesondere „sozial- und gesellschaftspolitische Bewusstseinsbildung“ und „Partizipation“ Kompetenzen sind, „die sich heute durch gruppendynamisches Lernen vermitteln“ (zu den Lernpotenzialen in gruppendynamischen Lern-Formaten vgl. auch Claußen 2015).

Es wäre insofern lohnenswert, vertieft zu diskutieren, was Krainz (2020, S. 491) für gruppendynamische Trainings und Beratung über das „Potenzial einer Widergewinnung des Politischen durch die Krise“ noch ohne Bezug zur Corona-Zeit beschreibt: Angesichts der Zeit-bezogenen Beobachtungen zu den Erfahrungen von Jugendlichen in der Corona-Pandemie könnten gruppendynamische Lernformate bei der Gestaltung von Aushandlungsorten für gesellschaftliche Mitgestaltungsmöglichkeiten für und mit jungen Menschen, sowie für das gegenseitige Verstehen und Vermitteln generationaler Erfahrungen in der Pandemie, einen wertvollen Beitrag leisten.

## Footnotes

1 Einhergehend mit der in Kap. 3 dargestellten Vielfalt der Bedeutungen des Begriffs der „Jugend“, werden die Bezeichnungen „junge Menschen“ und „Jugendliche“ in der Literatur nicht einheitlich verwendet. Um kenntlich zu machen, dass Jugend aus einer soziologischen Sichtweise nicht mit dem Erreichen eines bestimmten biologischen Alters endet, werden die beiden Begriffe im vorliegenden Artikel alternierend verwendet. Insbesondere soll die Bezeichnung „junge Menschen“ kenntlich machen, dass bspw. auch Schüler:innen und Student:innen über 20 Jahre, die im Begriff sind, jugendtypische Entwicklungsaufgaben zu bewältigen, hier „mitgemeint“ sind. Diese Begriffsverwendung ist insbesondere in sozialpädagogischen Diskursen verbreitet (vgl. z. B. Mangold 2016).

2 Zugunsten der besseren Lesbarkeit wurden die Originalzitate sprachlich geglättet und orthografische Fehler korrigiert.
